# A Theoretical Framework for Ligand-Functionalised Magnetic Lipid Nanoparticles in Glioblastoma Therapy

**DOI:** 10.3390/cancers17243905

**Published:** 2025-12-06

**Authors:** Dian Buist, Hiska van der Weide, Steven Bergink, Roland Chiu

**Affiliations:** 1 University College Groningen, University of Groningen, 9718 BG Groningen, The Netherlands; d.buist2004@gmail.com (D.B.); s.bergink@rug.nl (S.B.); 2 Department of Radiation Oncology, University of Groningen, University Medical Center Groningen, 9713 GZ Groningen, The Netherlands; h.l.van.der.weide@umcg.nl

**Keywords:** glioblastoma, blood-brain barrier, brain-tumour barrier, lipid nanoparticles, superparamagnetic iron oxide nanoparticles, magnetic hyperthermia, ligand functionalisation, targeted drug delivery, tumour microenvironment

## Abstract

Glioblastoma multiforme (GBM) is a highly aggressive primary brain tumour with limited treatment options and a poor prognosis. Therapeutic failure is driven by multiple barriers, including the blood–brain barrier (BBB), the tumour microenvironment (TME), and intratumoural heterogeneity. Conventional delivery systems often fail to achieve sufficient drug accumulation or controlled release within the tumour. In this review, we outline a theoretical framework for the design of ligand-functionalised magnetic lipid nanoparticles (MF-R-LNs), a multifunctional nanoplatform that integrates active targeting, stimuli-responsive drug release, and external magnetic-field control. The proposed MF-R-LNs incorporate superparamagnetic iron oxide nanoparticles (SPIONs) for magnetic guidance and hyperthermia; polyethylene glycol (PEG) for extended circulation; and surface ligands such as peptides, antibodies, or aptamers to target GBM-specific receptors including epidermal growth factor receptor (EGFR), Interleukin-13 receptor alpha-2 (IL-13Rα2), and integrins. Triggered release mechanisms such as pH-sensitive lipids, redox cleavable linkers, and enzyme-responsive coatings enable selective drug release within the TME. Magnetic hyperthermia serves as both a therapeutic modality and a remote trigger to enhance release and tumour penetration. This modular design offers a theoretically robust strategy to overcome the key physiological and therapeutic barriers in GBM. We discuss the rationale behind each design feature, explore potential synergies, and highlight translational challenges such as tumour heterogeneity, manufacturing complexity, and safety concerns. Despite encouraging preclinical evidence, clinical translation faces substantial hurdles, notably patient-specific heterogeneity and scalable GMP manufacturing/characterisation of multi-component nanoplatforms. While preclinical validation remains necessary, this framework may inform future efforts to develop spatiotemporally controlled, multifunctional therapeutics for glioblastoma. This manuscript is a conceptual framework review that synthesises current strategies into actionable guidance for designing and reporting MF-R-LNs for GBM.

## Simple Summary

Glioblastoma (GBM) is hard to treat because most drugs cannot cross the blood–brain barrier and the tumour rapidly develops resistance. We outline a practical design blueprint for ligand-functionalised, magnetic lipid nanoparticles that can circulate longer, bind to GBM-specific receptors, and release their cargo only inside the tumour microenvironment. By embedding superparamagnetic iron oxide nanoparticles (SPIONs), the platform also enables external magnetic guidance and mild hyperthermia to trigger on-demand drug release. We describe how to choose ligands, tune size and surface chemistry, and report magnetic-field parameters safely and reproducibly. The aim is to give researchers and clinicians a clear, modular framework that integrates known components into a single strategy to improve delivery and efficacy in GBM.

## 1. Introduction

Glioblastoma (GBM) remains the most aggressive primary brain tumour in adults, with survival limited by poor drug penetration and rapid therapeutic resistance [1,2]. Standard of care therapy includes surgical resection, radiotherapy, and temozolomide chemotherapy, yet tumour recurrence is nearly universal [3]. Multiple barriers—including the blood–brain barrier (BBB), a heterogeneous blood–tumour barrier (BTB), and a hostile tumour microenvironment (TME)—undermine delivery [4]. Here, we outline a practical design framework for ligand-functionalised, magnetic lipid nanoparticles (MF-R-LNs) and propose clear reporting conventions for magnetic-field use in GBM.

One of the most significant obstacles to effective GBM therapy is the BBB, a tightly regulated interface that prevents many therapeutic agents from reaching the brain parenchyma [5]. Even in areas where the BBB is compromised, referred to as the BTB, drug delivery remains highly variable and incomplete [6]. The TME further complicates treatment due to hypoxia, acidic pH, aberrant vasculature, and a heavily immunosuppressive milieu. Interstitial fluid pressure (IFP) is typically 0–2 mmHg in normal brain but often 10–30 mmHg in GBM, with peritumoural regions reported up to 40–50 mmHg, further impeding convective drug transport. Compounding these factors are therapy-induced resistance mechanisms such as upregulation of efflux transporters, DNA repair enzymes such as O6-Methylguanine-DNA Methyltransferase (MGMT), and activation of redundant signalling pathways [7]. Recent GBM nano approaches such as intranasal small-molecule formulations (e.g., NEO100); catheter-based radionanoliposomes (e.g., 186RNL); intralesional magnetothermal superparamagnetic iron oxide nanoparticles (SPIONs) (e.g., NanoTherm); and BBB-shuttle carriers (e.g., glutathione- or Angiopep-based), demonstrate feasibility but each addresses only part of the problem. This motivates systemically deliverable platforms that can reach tumour, be guided/monitored, and release on-target. We therefore introduce MF-R-LNs as a coherent design space built around these three aims.

To overcome these challenges, there has been growing interest in developing multifunctional nanocarrier systems that can both traverse the BBB and selectively release their payload within the GBM microenvironment [8]. Lipid nanoparticles (LNPs) have emerged as versatile platforms due to their biocompatibility, ability to encapsulate hydrophilic and hydrophobic drugs, and potential for surface functionalisation. Magnetic nanoparticles, particularly SPIONs, offer an additional dimension of spatial control through magnetic guidance and the possibility of inducing hyperthermia under alternating magnetic fields; the resultant local heating of 42–45 °C fluidizes thermosensitive lipid bilayers and permeabilizes endosomal membranes, hence initiating on-demand drug release.

This review outlines a theoretical framework for designing ligand-functionalised, MF-R-LNs tailored to the therapeutic needs of GBM [9,10]. By integrating targeting ligands, PEGylation, redox- or enzyme-sensitive linkers, and magnetic cores, these multifunctional constructs aim to bypass physical barriers, enhance tumour specificity, and trigger localised drug release. The rationale for each design component is grounded in GBM pathophysiology and prior nanomedicine research, with emphasis on modularity and combinatorial synergy.

In addition to reviewing the rationale and design considerations, we explore how magnetic hyperthermia, activated remotely via SPIONs, can act as a secondary trigger to enhance intratumoural drug delivery and sensitise resistant tumour regions. We discuss potential applications of these theoretical systems in overcoming key limitations of current GBM therapy, as well as the translational hurdles that remain, including safety, ligand receptor heterogeneity, and manufacturing scalability.

By proposing a cohesive strategy that combines BBB penetration, microenvironment responsiveness, and magnetic control, this conceptual framework contributes to the growing discourse on next-generation nanotherapeutics for glioblastoma. While experimental validation remains a future goal, the principles outlined here may help guide the rational design of multifunctional delivery systems with improved therapeutic efficacy and translational potential.

## 2. Barriers to Drug Delivery in Glioblastoma

**Figure 1:**
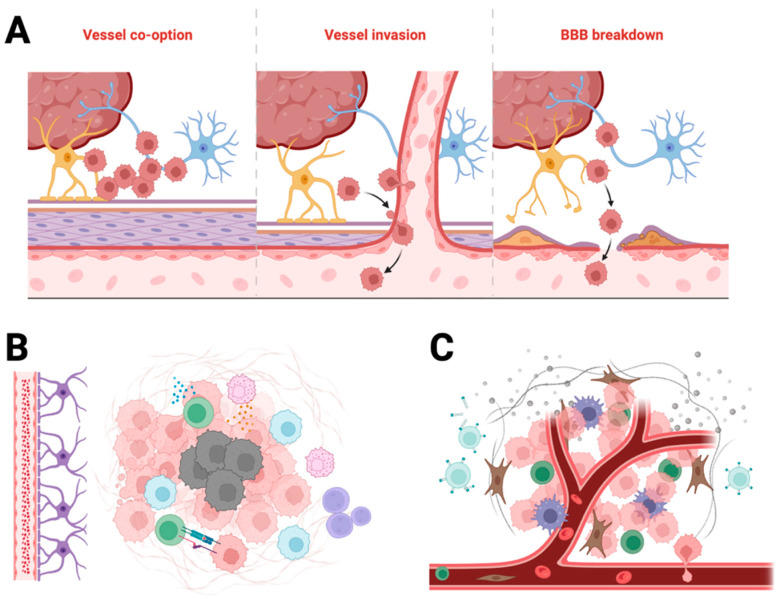
Barriers to drug delivery in glioblastoma. Schematic overview of barriers that limit therapeutic delivery to GBM. (**A**) Vascular barriers: the intact blood–brain barrier (BBB) and heterogeneous blood–tumour barrier (BTB) restrict parenchymal entry; high efflux via P-glycoprotein (P-gp) and breast cancer resistance protein (BCRP) further lowers intratumoural drug levels. (**B**) Physical and chemical microenvironment: dense extracellular matrix (ECM) and elevated interstitial fluid pressure (IFP) impede convection and diffusion, while hypoxia and acidic pH reduce drug efficacy and promote resistance. (**C**) Immunological and evolutionary barriers: tumour-associated macrophages (TAMs), myeloid-derived suppressor cells (MDSCs) and regulatory T cells (Tregs) sequester nanoparticles and dampen antitumour responses; genomic heterogeneity and pathway redundancy (e.g., MGMT, EGFR/PI3K/AKT) drive adaptive resistance over time. Visual conventions: neurons (blue), astrocytes (purple), endothelial cells with tight junctions (pink), pericytes (tan), red blood cells (red), cancer cells (rose) and immunosuppressive populations—TAMs/MDSCs/Tregs (green tones).

Despite decades of research, GBM remains one of the most treatment-refractory cancers. Its resistance to therapy arises from a confluence of structural, cellular, and molecular barriers that impair drug delivery and promote tumour survival. Understanding these barriers is essential for rational design of nanomedicines capable of penetrating the tumour, overcoming resistance mechanisms, and delivering payloads effectively. These features are illustrated in Figure 1, which summarises the structural and physiological barriers that restrict drug delivery to GBM.

### 2.1. The Blood-Brain Barriers and Blood-Tumour Barrier

The BBB is a selective endothelial interface formed by tight junctions, pericytes and astrocytic end-feet that restrict the entry of most systemic drugs into the central nervous system [11,12]. Although the BBB may be locally disrupted in GBM, the resulting BTB remains heterogeneously permeable. This regional variability causes uneven drug distribution, with poorly perfused tumour regions remaining inaccessible to most small molecules and biologics.

Furthermore, endothelial cells in the BBB and BTB express high levels of efflux pumps such as P-glycoprotein (P-gp) and breast cancer resistance protein (BCRP), which actively transport chemotherapeutic agents back into the circulation [13]. This severely limits the brain uptake of many systematically administered drugs and contributes to subtherapeutic concentrations in the tumour core.

### 2.2. Tumour Microenvironment (TME)

The GBM microenvironment presents additional biophysical and biochemical barriers. The extracellular matrix is highly dense and irregular, impeding nanoparticle penetration [14]. Hypoxia and necrosis are common, creating an acidic and immunosuppressive milieu that supports angiogenesis, tumour invasion, and resistance to radiotherapy and chemotherapy.

Tumour-associated macrophages (TAMs), myeloid-derived suppressor cells (MDSCs), and regulatory T cells (Treg) further contribute to immune evasion and therapeutic resistance. These immune cells can also internalise drug carriers nonspecifically, reducing delivery efficiency to glioma cells.

### 2.3. Therapy-Induced Resistance Mechanisms

GBM is characterised by rapid mutation rates and high intratumoural heterogeneity. As a result, subclonal populations with therapy-resistant phenotypes frequently emerge. Upregulation of DNA repair enzymes such as MGMT confers resistance to alkylating agents such as temozolomide [12,15]. Parallel activation of redundant signalling pathways (e.g., EGFR, PI3K/AKT/mTOR) enables escape from targeted therapies [12,13].

Additionally, the adaptive expression of efflux transporters and anti-apoptotic proteins under therapeutic pressure further reduces the effectiveness of chemotherapeutics [13,16]. The dynamic and adaptable nature of GBM requires delivery systems that can not only reach the tumour but also remain effective in the face of evolving resistance mechanisms.

## 3. Design Framework for Ligand-Functionalised Magnetic Lipid Nanoparticles

The design of multifunctional nanoparticles for GBM therapy requires strategic integration of features that address key delivery challenges: crossing the BBB, targeting tumour cells, avoiding immune clearance, and triggering drug release selectively within the TME [12]. Ligand-functionalised MF-R-LNs are conceptual platforms that unite several design principles to achieve this goal.

### 3.1. Core Structure and Composition

**Figure 2:**
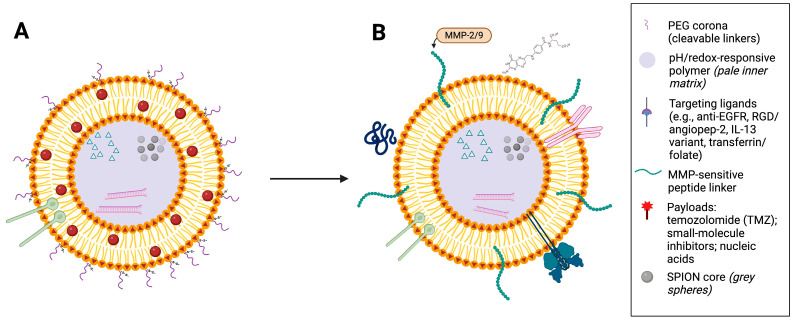
Stealth-to-activated ligand-functionalised magnetic lipid nanoparticle (MF-R-LN). (**A**) Stealth state: lipid-bilayer carrier with a PEG corona, embedded superparamagnetic iron oxide nanoparticle (SPION) core, targeting ligands, and encapsulated payload. (**B**) Activated state after tumour accumulation: tumour-associated enzymes (e.g., MMP-2/9) and intracellular pH/redox cues reduce PEG shielding and promote stimulus-triggered release; the SPION core enables magnetic guidance and mild hyperthermia. In (**B**), surface features are shown generically—targeting ligands attached via MMP-sensitive linkers with partially shed PEG—while a pale inner matrix denotes a pH/redox-responsive polymer; grey spheres indicate the SPION core, and payloads are depicted inside.

The foundational structure of MF-R-LNs is a lipid-based carrier, selected for its biocompatibility, ability to encapsulate both hydrophobic and hydrophilic drugs, and ease of surface modification [7,17]. The lipid bilayer can be formulated to include phase-transition lipids or thermosensitive elements that respond to external triggers such as heat or magnetic fields [18]. A representative design of a ligand-functionalised MF-R-LN is shown in Figure 2.

In the nanoparticle core, superparamagnetic iron oxide nanoparticles (SPIONs) are embedded to enable magnetic steering and hyperthermia. SPIONs are well characterised and have been used clinically in diagnostic and therapeutic contexts [9]. Their inclusion allows for both passive and active guidance across the BBB and for localised activation using an alternating magnetic field [19].

Polyethylene glycol (PEG) is commonly grafted onto the nanoparticle surface to reduce opsonization by the mononuclear phagocyte system and extend systemic circulation time. While PEGylation improves stealth properties, it may reduce cell uptake, necessitating the inclusion of targeting ligands to compensate [20,21,22].

### 3.2. Surface Functionalisation and Targeting Ligands

Surface functionalisation with targeting ligands enhances selectivity toward tumour-specific receptors overexpressed in GBM. Candidate ligands include peptides (e.g., angiopep-2, RGD) [23,24,25], antibodies (e.g., anti-EGFR), aptamers, or small molecules such as folate. These ligands can be conjugated via stable or cleavable linkers depending on the desired release profile and microenvironmental triggers.

Receptor targets must be chosen based on expression patterns in GBM cells versus healthy brain tissue. Ideal targets include the following:

1. EGFR and EGFRvIII: commonly overexpressed in IDH-wildtype tumours and associated with poorer prognosis
2. IL-13Rα2: high specificity to GBM cells
3. Integrins (e.g., αvβ3, αvβ5): involved in tumour angiogenesis and invasion
4. Transferrin receptor (TfR): facilitates BBB crossing [26]

Given marked inter- and intra-patient heterogeneity, ligand choice should be patient-informed wherever feasible, via biopsy immunophenotyping (e.g., receptor panels for LRP1, TfR, EGFR/EGFRvIII, IL-13Rα2) or liquid-biopsy surrogates (circulating tumour DNA/exosomes), together with imaging biomarkers. In practice, a dual-ligand design that pairs a BBB-transcytosis shuttle (e.g., Angiopep-2 or a TfR-directed binder) with ≥1 tumour-binding ligand selected from the patient’s profile helps preserve target engagement despite spatial heterogeneity and temporal evolution.

Table 1 summarises representative ligand–receptor pairs relevant to GBM. Ligand selection should balance affinity and specificity: very high-affinity binders risk off-target binding and sink effects, whereas low-affinity binders may yield insufficient uptake. Marked heterogeneity within and between tumours further motivates redundancy (e.g., dual-ligand builds) and periodic reassessment as tumours evolve

### 3.3. Stimuli-Responsive Release

MF-R-LNs can be engineered to release their payload in response to specific stimuli within the TME or external triggers. Common mechanisms include the following:

1. pH sensitive lipids or polymers: exploit acidic tumour pH for membrane destabilisation;
2. Redox sensitive linkers (e.g., disulfide bonds): cleaved by elevated intracellular glutathione [54];
3. Enzyme-responsive elements: cleavable by matrix metalloproteinases (e.g., MMP-2/9), which are elevated in GBM during invasive growth, angiogenesis, and hypoxia-driven ECM remodelling; note that MMPs also rise in inflammatory CNS disorders (e.g., MS), so pair this trigger with tumour-targeting ligands or intracellular cues to preserve specificity [55,56];
4. These mechanisms increase spatiotemporal control of drug delivery, reducing systemic toxicity and enhancing local therapeutic effect.

### 3.4. Practical Constraints and Manufacturability

The modular nature of MF-R-LNs allows each functional element (core, coating, targeting ligand, release mechanism) to be tailored for a given therapeutic context. This platform can carry diverse payloads, including chemotherapeutics, gene agents (e.g., siRNA or CRISPR/Cas9 components), and immunomodulators. It also supports personalised designs for GBM heterogeneity by combining a BBB-transcytosis shuttle with at least one tumour-binding ligand and layered, stimulus-responsive release. Where profiling is limited or unavailable, a dual-ligand configuration provides a pragmatic default.

Therapeutic designs must also consider practical constraints, including the following:

1. Manufacturability and scale-up. Reproducibility across batches is essential; detailed critical quality attributes (CQAs) and assays are outlined in Section 6.2.
2. Hydrodynamic size and surface charge. A practical window of 80–150 nm with near-neutral to mildly negative ζ-potential can favour BBB interaction while limiting rapid reticuloendothelial system (RES) clearance [43].
3. Colloidal and oxidative stability/leakage control. Use cholesterol-stabilised lipid blends (avoid storage/handling near the bilayer Tm) and validate leakage at 37 °C and under alternating magnetic fields (AMF); consider antioxidant lipids/additives where warranted.
4. Core passivation and iron control. Prioritise passivated SPION cores (e.g., silica/dextran/phosphonate shells or equivalent) and minimise labile iron to mitigate Fenton-type chemistry (see Section 6.3).
5. Regulatory and safety profiles. Component-level identity/purity/stability criteria and device documentation are required; AMF reporting conventions are summarised in Section 6.4.

Detailed good manufacturing practice/chemistry, manufacturing and controls (GMP/CMC), safety/immunogenicity, and regulatory/AMF reporting requirements are provided in Section 6.2, Section 6.3 and Section 6.4 (CQAs; PEG immunogenicity; SPION characterisation and MRI/MPI tracking; AMF parameters H, f, H × f, thermometry, calibration).

With these constraints defined, we next consider magnetic hyperthermia as a trigger, how SPION-mediated heating can be harnessed to enable controlled, on-target drug release (Section 4).

## 4. Magnetic Hyperthermia as a Trigger

Magnetic hyperthermia is a promising adjunctive modality for enhancing drug delivery to GBM by enabling externally controlled, localised heating within the tumour. When exposed to an AMF, SPIONs embedded in lipid nanoparticles generate heat through Néel and Brownian relaxation. This localised thermal effect can be exploited in multiple ways to improve outcomes.

### 4.1. Heat Induced Drug Release

**Figure 3:**
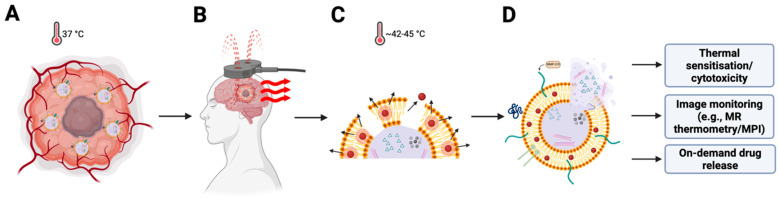
Alternating-magnetic-field triggering of heating and release from MF-R-LNs. (**A**) Intratumoural accumulation of heat-responsive, ligand-functionalised magnetic lipid nanoparticles (MF-R-LNs) at physiological temperature (37 °C). (**B**) Application of an alternating magnetic field (AMF) over the tumour region. (**C**) Superparamagnetic iron oxide nanoparticle (SPION) cores dissipate heat (42–45 °C) via Néel/Brownian relaxation, transiently perturbing the lipid bilayer and promoting membrane permeabilisation/endosomal escape. (**D**) Stimulus-triggered payload release into tumour cells and the microenvironment enables thermal sensitisation/cytotoxicity, image monitoring with conventional MRI (e.g., T2/FLAIR) or magnetic particle imaging (MPI), and dose-titration with MR thermometry during AMF, with on-demand drug release.

Thermosensitive lipids incorporated into the MF-R-LN bilayer can be designed to undergo phase transitions slightly above physiological temperatures (e.g., 42–45 °C) [18,57,58]. Upon AMF activation, SPION-mediated heating destabilises the membrane and triggers controlled drug release, enabling temporal precision so that release can be synchronised with tumour localisation (Figure 3). Typical alternating-magnetic-field parameters for SPION hyperthermia are 100–500 kHz at <15 kA·m^−1^, with safety limits applied to avoid non-specific tissue heating.

Formulation choices should minimise baseline leakage at 37 °C while preserving a sharp release transition at the target temperature. In practice, this often requires blended lipid compositions with cholesterol for bilayer stabilisation, rather than relying on a single saturated lipid (e.g., DPPC alone). Optional polymeric over-layers (e.g., PNIPAM) can narrow the thermal window but add complexity and should be justified by leakage and release assays under physiologic and hyperthermic conditions. Overall, lipid composition, cholesterol fraction, ligand density, and particle size should be jointly tuned against stability vs. triggerability criteria established in vitro before in vivo use.

### 4.2. Tumour Sensitisation and Direct Cytotoxicity

Magnetic hyperthermia may also contribute directly to tumour cell death via local heating. Even moderate hyperthermia (42 °C) can sensitise tumour cells to chemotherapy and radiotherapy by disrupting DNA repair pathways, increasing membrane permeability, and promoting apoptosis; inducing a local heat shock response [58,59]. At higher temperatures, thermal ablation can occur, although this requires more energy and may risk damaging surrounding healthy tissue.

GBM’s dense extracellular matrix and irregular vascularization often create poorly perfused regions that are resistant to therapy. Hyperthermia may improve perfusion and oxygenation in these hypoxic zones, thereby enhancing drug penetration and overall treatment efficacy [60].

### 4.3. Design Considerations for Magnetic Responsiveness

Effective magnetic hyperthermia requires careful optimisation of SPION characteristics and field conditions:

1. Size and shape. Cores of approximately 10–20 nm remain superparamagnetic and minimise aggregation [61,62]; narrow polydispersity is preferred.
2. Field parameters. Always report the field amplitude H (kA·m^−1^), frequency f (kHz), their product H × f, and exposure duration, and target mild hyperthermia 42–45 °C at the lesion with verified thermometry.
3. Surface modification. Biocompatible coatings reduce aggregation and opsonisation and improve colloidal stability and safety.
4. Hybrid formulation. Embedding SPIONs within lipid matrices requires uniform dispersion and sustained stability [62,63]; lipid–magnetic hybrids enable guidance and stimulus-triggered release.

Magnetic-field safety. A commonly cited whole-body comfort heuristic is H × f ≤ 4.85 × 10^8^ A·m^−1^·s^−1^ (Atkinson–Brezovich). Focal, brain-targeted applications may operate above this with careful temperature monitoring; for example, clinical SPION hyperthermia has used H ≈ 18 kA·m^−1^ and f ≈ 100 kHz (H × f ≈ 1.8 × 10^9^ A·m^−1^·s^−1^). As a practical upper bound, many groups remain below 5 × 10^9^ A·m^−1^·s^−1^ (Hergt–Dutz heuristic). In all cases, report the measured intratumoural temperature and the measurement method; numeric heuristics never replace thermal safety monitoring [61,64].

### 4.4. Advantages of Remote Control

The ability to remotely control both nanoparticle localisation and drug release distinguishes MF-R-LNs from conventional delivery systems. By applying an external magnet or AMF, clinicians may enhance tumour accumulation via magnetic targeting and initiate payload release only after tumour localisation. This two-step strategy offers potential improvements in both selectivity and safety, reducing off-target toxicity and system side effects.

## 5. Translation and Clinical Outlook

The true therapeutic potential of ligand-functionalised MF-R-LNs lies not in any single design feature, but in the strategic integration of multiple functionalities that address distinct challenges in GBM treatment. By combining passive and active targeting, stimuli-responsive release, magnetic guidance, and hyperthermia, these nanoparticles can theoretically achieve a level of spatiotemporal precision unattainable with conventional therapies or single function nanocarriers.

This level of spatiotemporal control permits the cautious use of otherwise prohibitive, highly cytotoxic agents, which is attractive in a therapy-resistant disease such as GBM. Magnetic guidance provides receptor-agnostic localisation, concentrating the formulation at the lesion even when target receptors are sparse or heterogeneous, while ligands and tumour-specific triggers (e.g., MMP-sensitive linkers, pH/redox cues) retain cellular specificity and govern on-demand release. Crucially, localisation and delivery are monitorable: the magnetic core is visible on standard brain MRI (typically as focal hypointensity on T2/FLAIR) or via magnetic particle imaging (MPI); during AMF exposure, MR thermometry can titrate sessions to keep intratumoural peaks within 42–45 °C. In combination, guidance + imaging + triggered release create a pragmatic, closed-loop workflow not achievable with receptor-only “smart” drugs.

### 5.1. Integration of Targeting and Triggered Release

Surface ligands enhance cellular uptake by targeting overexpressed receptors on GBM cells and endothelial cells at the BBB. However, uptake alone does not guarantee therapeutic success, the cargo must be released intracellularly, ideally in response to tumour-specific cues. MF-R-LNs enable this through pH-, redox-, or enzyme-sensitive mechanisms, combined with externally applied magnetic hyperthermia to fine tune release kinetics.

The synergy between active targeting and stimuli-triggered release reduces premature leakage, improves tumour localisation, and increases intratumoural drug concentration, particularly in poorly perfused or hypoxic regions. The addition of magnetic guidance further enhances this selectivity by allowing non-invasive control over nanoparticle distribution.

### 5.2. Multimodal Therapeutic Potential

While chemotherapy is the most obvious payload for MF-R-LNs, the platform is inherently flexible and can accommodate the following:

1. Gene therapies (e.g., siRNA or plasmids targeting MGMT or EGFR);
2. Immunomodulators (e.g., STING agonists or checkpoint inhibitors);
3. Photothermal or radiotherapy sensitisers [2].

Multimodal therapy is especially relevant in GBM, where redundancy in survival pathways allows tumours to evade monotherapies. A single, modular system that co-delivers drugs and gene-silencers and releases them under tightly controlled conditions offers a rational response to tumour heterogeneity. With image guidance, otherwise prohibitive cytotoxic agents can be used more safely: magnetic guidance provides receptor-agnostic localisation, and the magnetic core is imageable (MRI/MPI), with MR thermometry during AMF to titrate exposure. See Table 1 for ligand options that support this strategy.

### 5.3. Personalised and Adaptive Design

Because the MF-R-LN system is modular, its components can be personalised based on the molecular profile of an individual tumour. Ligand selection can be tailored to receptor expression, and release mechanisms can be adapted depending on microenvironmental conditions such as MMP levels or redox gradients. This adaptability could support personalised medicine approaches in GBM, where patient-to-patient variation is profound.

Future developments may also include “smart” feedback systems, in which drug release is dynamically adjusted in response to local temperatures, pH, or metabolic changes detected in real-time.

Where feasible, a minimal molecular profile—EGFR/EGFRvIII, IL-13Rα2, integrins αvβ3/αvβ5, and transcytosis targets (TfR/LRP1) by IHC or targeted RNA/NGS—can prioritise ligand selection. Map results to Table 1; if expression is low, heterogeneous, or unavailable, proceed without delay using a dual-ligand design (BBB shuttle + tumour-binding ligand) with redundancy.

### 5.4. Clinical Feasibility

In practice, early studies would prioritise recurrent GBM with supratentorial, measurable lesions away from eloquent cortex, managed on stable steroids/anticonvulsants. MF-R-LN infusion could be scheduled between radiotherapy fractions or in combination with temozolomide at recurrence, with one to three AMF sessions per cycle depending on thermal readouts. Safety monitoring would include neurological exam, adverse-event surveillance, and temperature limits to keep intratumoural peaks within 42–45 °C. MR thermometry (where available) or predefined surrogate triggers (time-at-field, SAR estimates) would gate session duration. These practical constraints inform dose escalation and scheduling.

In practice, first-in-human evaluation would prioritise recurrent/refractory GBM under a Phase 0/1 framework with image-guided localisation (conventional MRI/T2–FLAIR hypointensity or MPI) and MR-thermometry–titrated AMF, with standard field reporting (H, f, H × f).

## 6. Translational Barriers and Limitations

While the conceptual design of MF-R-LNs offers an elegant solution to many of glioblastoma’s therapeutic challenges, several key limitations must be addressed before clinical translation can be realised. These include biological variability, engineering complexity, safety concerns, and regulatory barriers.

Clinical translation of MF-R-LNs will depend on five interconnected domains: tumour heterogeneity and personalisation, safety/immunogenicity, scalable GMP/CMC, regulatory pathway and trial design, and the limitations of current preclinical models. In this section, we outline the principal risks and practical mitigations for each domain, with emphasis on assayable quality attributes, reporting conventions for magnetic-field use, and study designs proportional to risk in recurrent GBM. These considerations are intended as design constraints and research priorities, not solved problems.

### 6.1. Ligand Specificity and Tumour Heterogeneity

Targeting ligands are critical for enhancing nanoparticle specificity and uptake, but their effectiveness is constrained by the heterogeneous nature of glioblastoma. Receptor expression varies widely not only between patients but also within tumour subregions, making it unlikely that a single ligand will ensure comprehensive targeting [65]. Over reliance on one receptor system may leave large fractions of the tumour unaddressed, particularly in invasive margins where expression profiles differ from the tumour core.

Multivalent or dual-ligand strategies may mitigate this issue, but at the cost of increased formulation complexity and potential off-target interactions [66]. Moreover, receptor saturation or downregulation following repeated exposure could reduce efficacy over time.

### 6.2. Manufacturing and Scale-Up

Each MF-R-LN component (lipid matrix, SPION core, PEGylation, ligand conjugation) introduces added synthesis and quality control requirements, and maintaining consistent size, surface charge, and ligand density is challenging at scale. Magnetic nanoparticles are prone to aggregation and require stabilising surface chemistries to remain colloidally stable in biological media; embedding them within lipid matrices without compromising bilayer integrity or inducing payload leakage necessitates careful optimisation. Large-scale production must ultimately meet GMP standards, which remains difficult for multifunctional, modular nanocarriers [61,67].

Translation will require a defined GMP/CMC package with batch-to-batch control of size/PDI/ζ-potential, ligand density (per particle), PEG density/cleavability, payload loading and release kinetics, and SPION content/relaxivity, alongside sterility/endotoxin and stability testing. CQAs and assays should be prespecified—e.g., DLS/NTA/SEC-MALS for size distributions, LC–MS for linker identity, ICP-MS/magnetometry for iron content and properties, and MRI relaxometry/MPI for tracking—while prioritising closed, scalable steps (in-line filtration, low-bioburden buffers) with complete batch records.

### 6.3. Safety and Immunogenicity

Although lipid-based systems and SPIONs are individually well studied, their combined use in multifunctional constructs raises additional toxicological questions. The long-term fate of iron oxide in brain tissue under repeated AMF exposure remains uncertain, with risks of oxidative stress, iron overload, and localised overheating [68]. Surface passivation, core size/valence control, and dosing strategies should be used to mitigate Fenton-type chemistry and unintended tissue effects.

PEGylation, once considered inert, can elicit anti-PEG antibodies, accelerating clearance or provoking hypersensitivity reactions [20]. Alternative stealth coatings, lower or cleavable PEG densities, and prospective immunosurveillance (e.g., anti-PEG titres) may reduce risk.

For uses involving alternating magnetic fields, authors should report H (kA·m^−1^), f (kHz), H × f, exposure duration, and in situ thermometry to manage heating risk, and include device calibration/phantom data to document field uniformity and temperature accuracy.

### 6.4. Regulatory and Clinical Pathways

Multifunctional nanomedicines are often regulated as combination products. Regulators will expect clear identity, purity, and stability criteria for each functional element (core, coating, linkers, ligands, payload) and for the assembled particle; validated release, immunogenicity, and safety assays; and predefined stopping rules for device-related heating. Even when components such as PEG, SPIONs, and phospholipids are individually familiar, the assembled construct carries additional burden due to potential synergy and emergent behaviour [17,61]. Device calibration/phantom documentation should accompany clinical use to demonstrate field uniformity and temperature accuracy.

Early clinical evaluation should prioritise recurrent/refractory GBM, where the risk–benefit is most favourable. A pragmatic Phase 0/1 approach can combine a small sentinel single-patient run-in with 3 + 3 or model-based dose escalation under Data and Safety Monitoring Board oversight. Co-primary endpoints are safety/tolerability and device/procedure feasibility; secondary readouts include intratumoural localisation (conventional MRI T2/FLAIR hypointensity or MPI), thermal-dose metrics via MR thermometry during AMF, PK/PD, and preliminary activity by Response Assessment in Neuro-Oncology (RANO).

A minimum preclinical package should precede first-in-human work: orthotopic GBM models for biodistribution, imageability, on-target heating, and efficacy; GLP toxicology (single and repeat dose), neurobehavioural testing, brain histopathology, iron fate/overload, and immunotoxicity (e.g., complement activation/anti-PEG); haemocompatibility/pyrogenicity assays; device–nanoparticle interaction testing (specific absorption rate, eddy-current effects, and H × f kept within practical heuristics); and, where feasible, large-animal studies for field dosimetry and skull-heating margins.

### 6.5. Magnetic-Field Applications and Clinical Infrastructure

Therapeutic use of magnetic fields for guidance and hyperthermia requires specialised equipment and trained teams. While MRI and some local-heating systems are widespread, adapting field generators for treatment remains niche and logistically demanding. Ensuring that therapeutic heating remains within safe, effective temperature ranges across heterogeneous tissues is non-trivial and may necessitate individualised calibration and on-line thermometry. Moreover, dedicated hyperthermia infrastructure is not yet standard in most oncology centres, which may limit access and scale-up, particularly in resource-limited settings [69].

## 7. Future Directions

**Figure 4:**
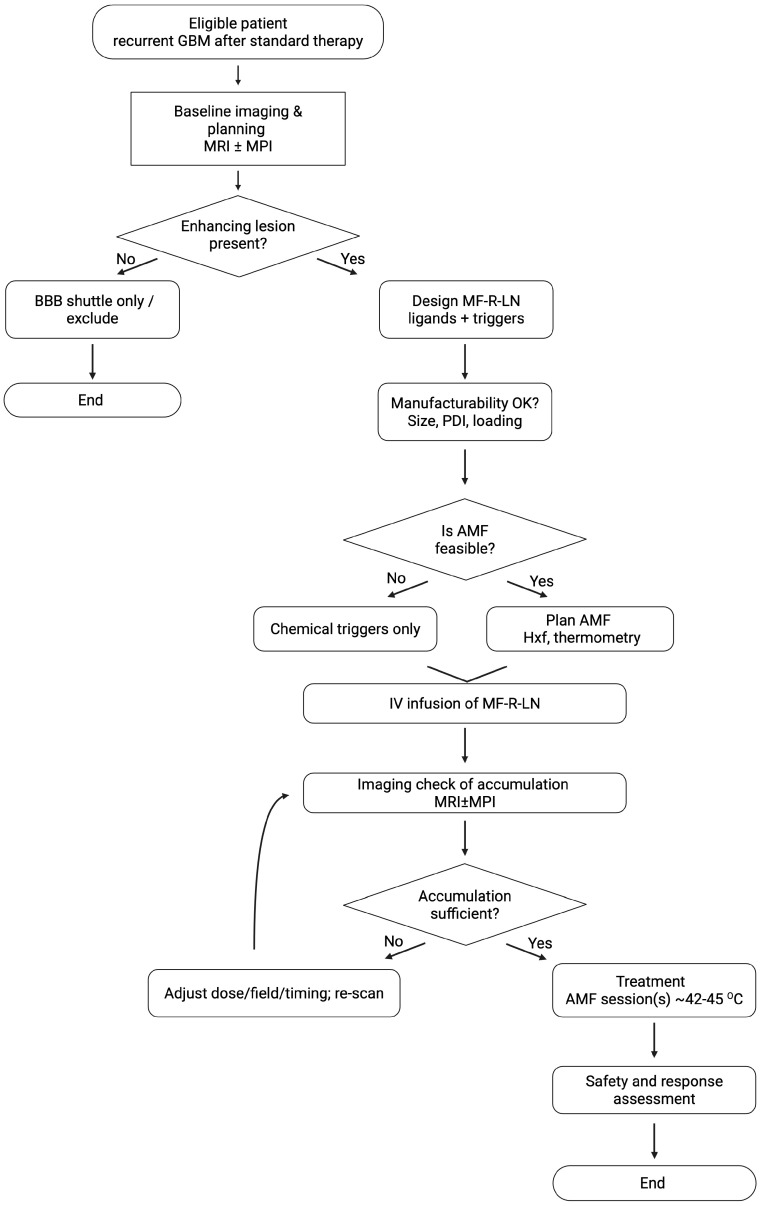
Clinical decision flow for MF-R-LNs in glioblastoma. Eligible patients (recurrent GBM) undergo baseline imaging and planning (MRI ± MPI). If a contrast-enhancing lesion is present, an MF-R-LN is designed (ligands by profile or dual strategy; pH/redox/MMP triggers) and its manufacturability confirmed (size, PDI, loading). Centre resources determine whether AMF can be used (within H × f comfort ranges with thermometry); if not, proceed with chemical triggers only. After IV infusion, an imaging check confirms intratumoural accumulation (MRI/MPI). If insufficient, adjust dose/field/timing and re-scan; if sufficient, proceed to treatment execution targeting mild hyperthermia (42–45 °C) with predefined monitoring and abort criteria, followed by safety and response assessment. A non-enhancing lesion may be managed with a BBB-shuttle-only approach or excluded from AMF-based protocols.

Although still theoretical, the MF-R-LN design offers a practical framework for overcoming the interlocking obstacles of GBM therapy. It points toward spatiotemporally controlled, multimodal treatment in which therapeutic agents are delivered with high specificity and released only where and when they are needed. By integrating well-established nanomedicine components into a coherent, modular platform, MF-R-LNs can guide experimental work and preclinical development. Importantly, the framework is not limited to glioblastoma: the principles of ligand targeting, magnetic responsiveness, and tumour-specific, stimuli-responsive release may extend to other solid tumours with similarly hostile microenvironments. The overall trajectory of the concept is summarised in Figure 4.

Future development should focus on precision ligand panels matched to patient-level receptor profiles; robust, modular self-assembly with controlled ligand density and iron loading; and real-time, closed-loop control using non-invasive imaging (e.g., MR thermometry or magnetic particle imaging). Interdisciplinary collaboration among materials science, neuro-oncology, and bioengineering will be essential to translate these designs into clinically viable treatments. In parallel, early engagement with regulators to align safety reporting (H, f, H × f, temperature) and manufacturing standards will reduce uncertainty. As regulatory frameworks for nanomedicine mature, MF-R-LNs could help define a new generation of tumour-specific, multifunctional therapeutics for glioblastoma and beyond.

## 8. Conclusions

GBM remains one of the most formidable challenges in oncology due to its invasive nature, resistance to therapy, and the physiological barriers that limit drug delivery to the brain. This review outlines a theoretical framework for multifunctional, ligand-functionalised MF-R-LNs as a strategy to address these challenges through targeted delivery, controlled release, and localised activation.

By integrating BBB-penetrating ligands, stimuli-responsive elements, and superparamagnetic cores, MF-R-LNs represent a rational and modular approach to overcome the major therapeutic barriers in GBM. These constructs offer spatiotemporal control over drug delivery, potential for synergistic multimodal therapy, and adaptability to personalised treatment strategies.

While experimental validation remains to be seen, the principles described here may guide future design and preclinical development of nanoparticle systems aimed at brain tumours and other difficult to treat solid malignancies. As advances in materials science, targeting strategies, and magnetic field technology continue to evolve, the vision of a precisely controllable, tumour-specific nanoplatform for glioblastoma therapy becomes increasingly feasible.

## Figures and Tables

**Table 1:** Ligand–receptor options for GBM targeting and practical considerations. Representative ligands with primary receptor/target, selectivity (GBM vs. normal brain), evidence (BBB/uptake), and pros/risks (practical). Conjugation chemistries and caveats are given in footnotes [a–c]. Payload examples are indicated by superscripts p1–p7 immediately after each ligand name in column 1. Clinical status/biocompatibility badges appear in the same cell as the ligand (e.g., [CNS-H], [Non-CNS-H], [APP-Non-CNS], [Pre-clin], [Low-immuno], [Immuno-flag]); see key below. Supporting references for each ligand are listed in the “References by ligand” table footer block.

Ligand	Primary Receptor/Target	Selectivity (GBM vs. Normal Brain)	Evidence (BBB/Uptake)	Pros/Risks (Practical)
Angiopep-2 ^p1^ [CNS-H] [Low- immuno]	LRP1	High on brain endothelium; variable on GBM cells; low on neurons	Preclinical BBB transcytosis; human CNS data	Good shuttle; peripheral LRP1 “sink”—tune ligand density; see [a]
Transferrin ^p2^ [Non-CNS-H] [Immuno-flag]	TfR/CD71	High on BBB endothelium and many tumours; present in normal tissues	Receptor-mediated BBB transcytosis (preclinical); human imaging/targeting outside CNS	Widely used shuttle; competition with endogenous Tf; avoid very high affinity/valency; see [a]
anti-EGFR (IgG/fragment) ^p3^ [APP-Non-CNS] [CNS-H] [Immuno-flag]	EGFR/EGFRvIII	Overexpressed in GBM (heterogeneous); minimal in normal brain	↑ cellular uptake; full IgG BBB-limited; fragments improve penetration	Potent binder; off-tumour EGFR (skin/gut); consider fragments/bispecifics; see [b]
IL-13 (variant/peptide) ^p4^ [CNS-H] [Immuno-flag]	IL-13Rα2	Frequent in GBM; minimal in normal brain; heterogeneous	Receptor-mediated internalisation; CNS human experience (selected agents)	GBM-selective axis; minimise IL-13Rα1 binding; species differences; monitor immunogenicity; see [b]
RGD/cRGD (peptide) ^p5^ [CNS-H] [Low-immuno]	Integrins αvβ3/αvβ5	Enriched on GBM cells/vasculature; low on normal brain endothelium	↑ tumour binding/penetration; not a BBB shuttle	Solid tumour targeting; platelet/vascular binding risk; prefer cyclic RGD; control multivalency; see [b]
Aptamers (e.g., AS1411) ^p6^ [Non-CNS-H] [Low-immuno]	Nucleolin (cell-surface)	Overexpressed on many tumour cells incl. GBM; low on normal brain surface	Receptor-mediated uptake in glioma models; BBB limited without shuttle	Small and engineerable; nuclease sensitivity—use 2′-mods/PEGylation; rapid renal clearance if small; see [c]
Folate (small molecule) ^p7^ [Non-CNS-H] [Low-immuno]	FOLR1 (± FRβ on macrophages)	Variable in GBM; low in normal parenchyma	Strong uptake in FR-positive cells; not a BBB shuttle	Simple chemistry; kidney uptake; competition with endogenous folate; heterogeneous expression; human experience outside CNS (late-phase trials), GBM use remains preclinical/early-phase; see [c].

[a] Angiopep-2/Transferrin: EDC/NHS (carboxyl–amine), maleimide–thiol via PEG spacer; Tf–PEG–lipid alternatives. [b] Antibodies/peptides: engineered cysteine/site-specific, maleimide–thiol, EDC/NHS; bispecific/fusion formats; PEG-spacers. [c] Aptamers/small molecules: 5′-amine/thiol, click (azide–alkyne), biotin–streptavidin, lipid-anchored conjugates; add 2′-mods for nuclease resistance. p1 (Angiopep-2): small-molecule chemotherapeutics; RNA therapeutics (siRNA/ASO); proteins/peptides. p2 (Transferrin): small-molecule chemotherapeutics; RNA therapeutics; proteins/peptides. p3 (anti-EGFR IgG/fragment): small-molecule inhibitors; RNA therapeutics targeting EGFR-axis; protein/biologic cargos. p4 (IL-13 variant/peptide): small-molecule chemotherapeutics; experimental toxin fusions; RNA therapeutics. p5 (RGD/cRGD): small-molecule chemotherapeutics; photosensitisers/imaging agents; RNA therapeutics (when co-displayed). p6 (Aptamers, e.g., AS1411): RNA/DNA aptamer payloads or conjugates; small-molecule cargos; imaging agents. p7 (Folate): small-molecule chemotherapeutics; imaging agents; selected RNA cargos via folate–PEG–lipid linkage. References by ligand: angiopep-2 [27,28,29,30,31,32]; RGD (cRGD) [23,33,34,35]; anti-EGFR [36,37,38]; aptamers (AS1411) [39,40,41]; folate [42,43,44,45]; IL-13 [24,46,47,48]; transferrin [49,50,51,52,53]. Clinical Status and Biocompatibility Key: [CNS-H] human data in brain/GBM; [Non-CNS-H] human data outside CNS; [APP-Non-CNS] approved in a non-CNS indication; [Pre-clin] preclinical only; [Low-immuno] generally low immunogenicity for class; [Immuno-flag] monitor for immunogenicity/hypersensitivity (class expectation or reports). Symbol: ↑, increased relative to non-targeted control.

## Data Availability

No new data were created or analysed in this study. Data sharing is not applicable to this article.

## Abbreviations

The following abbreviations are used in this manuscript:

BBB	Blood–Brain Barrier
BTB	Blood–Tumour Barrier
P-gp	P-glycoprotein
BCRP	Breast Cancer Resistance Protein
ECM	Extracellular Matrix
IFP	Interstitial Fluid Pressure
TAMs	Tumour-Associated Macrophages
MDSCs	Myeloid-Derived Suppressor Cells
Tregs	Regulatory T Cells
GBM	Glioblastoma
PEG	Polyethylene Glycol
SPION	Superparamagnetic Iron Oxide Nanoparticle
LRP1	Low-Density Lipoprotein Receptor-Related Protein-1
TfR	Transferrin Receptor
EGFR	Epidermal Growth Factor Receptor
FOLR1	Folate Receptor-α
AMF	Alternating Magnetic Field
MR	Magnetic Resonance
MPI	Magnetic Particle Imaging
MF-R-LN	Ligand-Functionalised Magnetic Lipid Nanoparticle
H × f	Field–Frequency Product
MRI	Magnetic Resonance Imaging
PDI	Polydispersity Index
